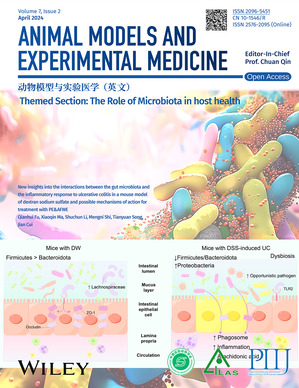# Cover Picture

**DOI:** 10.1002/ame2.12410

**Published:** 2024-05-08

## Abstract

The cover image is based on the article ‘New insights into the interactions between the gut microbiota and the inflammatory response to ulcerative colitis in a mouse model of dextran sodium sulfate and possible mechanisms of action for treatment with PE & AFWE’ (DOI: 10.1002/ame2.12405) reported by Qianhui Fu, Xiaoqin Ma, et al. The association between microbiota dysbiosis and the pathgenesis of IBD is complex and dynamic. When the intestinal ecosystem is in dysbiosis, the reduced abundance and diversity of intestinal gut microbiota make the host more vulnerable to the attack of exogenous and endogenous pathogenic gut microbiota.